# Synbiotic supplementation to decrease *Salmonella* colonization in the intestine and carcass contamination in broiler birds

**DOI:** 10.1371/journal.pone.0223577

**Published:** 2019-10-10

**Authors:** R. Shanmugasundaram, M. Mortada, D. E. Cosby, M. Singh, T. J. Applegate, B. Syed, C. M. Pender, S. Curry, G. R. Murugesan, R. K. Selvaraj

**Affiliations:** 1 Department of Poultry Sciences, University of Georgia, Athens, GA, United States of America; 2 USDA-ARS, Athens, GA, United States of America; 3 BIOMIN Holding GmbH, Getzersdorf, Austria; 4 BIOMIN America Inc., Overland Park, KS, United States of America; USDA-Agricultural Research Service, UNITED STATES

## Abstract

*In vitro* and *in vivo* experiments were conducted to study the effects of synbiotic supplementation on *Salmonella enterica* ser. Enteritidis (SE) proliferation, cecal content load, and broiler carcass contamination. *Lactobacillus reuteri*, *Enterococcus faecium*, *Bifidobacterium animalis*, and *Pediococcus acidilactici* culture supernatants decreased (P < 0.05) the *in vitro* proliferation of SE at 1:1 supernatant: pathogen dilution. A total of 240 Cobb-500 broiler chicks were randomly allotted to three treatment groups (8 replicates/group with 10 birds/replicate): control (basal diet), antibiotic (Virginiamycin at 20 mg/kg feed), synbiotic (PoultryStar^®^ ME at 0.5 g/kg feed containing *L*. *reuteri*, *E*. *faecium*, *B*. *animalis*, *P*. *acidilactici and a* Fructooligosaccharide) from day of hatch. At 21 d of age, all birds in experimental groups were orally inoculated with 250 μl of 1 X 10^9^ CFU SE. Antibiotic supplementation increased (P < 0.05) body weight and feed consumption, compared to the control group. Birds in the synbiotic supplementation had intermediate body weight and feed consumption that were not significantly different from both the control and antibiotic group at 42 d of age in SE infected birds. No significant effects were observed in feed efficiency at 42 d of age among the groups. Antibiotic and synbiotic supplementation decreased (P < 0.05) SE load in cecal contents by 0.90 and 0.85 log units/ g and carcass SE load by 1.4 and 1.5 log units/mL of rinsate compared to the control group at 42 d of age (21 dpi). The relative abundance of IL-10, IL-1, TLR-4, and IFNγ mRNA was decreased (P < 0.05) in the antibiotic and synbiotic supplementation groups compared to the control birds at 42 d of age (21 dpi). It can be concluded that synbiotic supplementation decreased SE proliferation *in vitro* and decreased SE load in the cecal contents and broiler carcass.

## Introduction

Salmonellosis is a foodborne illness, caused by the gram-negative enteric bacterium *Salmonella* and is of major public health importance in developing countries. The primary sources of human *Salmonella* infections are consumption of contaminated meat or eggs of *Salmonella*-positive chickens [1] and up to 9% of samples from poultry production can be positive for *Salmonella* [2]. Chicks acquire *Salmonella* via vertical transmission from parents and horizontal transfer from environmental facilities [3]. Most of the initial infection takes place early during post-hatch, although, *Salmonella* infection can occur during any part of the production cycle [4]. *Salmonella* control in poultry flocks is difficult since cleaning and disinfection fail to eliminate *Salmonella* in poultry [5]. Though HACCP (Hazard Analysis Critical Control Program) has reduced *Salmonella* contamination of chicken carcasses [6], recent multistate outbreak of multidrug-resistant- *S*. Heidelberg highlights the need to develop effective control measures to reduce *Salmonella* in the poultry industry [7].

In healthy humans, the *Salmonella* infectious dose is 10^6^ to 10^8^ [8], while chickens infected with *Salmonella* are persistent carriers [9]. *Salmonella* survives in the chicken intestine by inducing T regulatory cells (Tregs) [9]. Induced Tregs secrete Interleukin-10 (IL-10) and suppress the host immune responses, which could help *Salmonella* to escape host immune responses [9]. Virginiamycin is a commonly used antibiotic in poultry production and has been shown to affect *Salmonella* prevalence and abundance in poultry intestine [10].

*S*. *Enteritidis* is the predominant *Salmonella* serovar in human cases related to poultry contamination in US [11]. Numerous on-farm control strategies have been evaluated for control *Salmonella* shedding in poultry, including vaccination [12]. However, these control strategies have limited success in controlling *Salmonella* contamination in chicken [13], and hence, it is necessary to identify alternative on-farm strategies to control *Salmonella* infection in broilers.

Currently, the poultry industry applies probiotics and prebiotics to control issues associated with gut health. Probiotics are live fed microbial supplements and can maintain the microbial balance between beneficial and pathogenic bacteria in the gut [14] by producing antibacterial substances or through competitive exclusion by competing for attachment sites in the gut [15]. Prebiotics are non-digestible carbohydrates that act as a substrate for Bifidobacteria and lactic acid bacteria (LAB) in the colon [16]. Fructo-oligosaccharides, galacto-oligosaccharides, and mannan-oligoasacchardes are commonly applied as prebiotics in poultry production [17]. Prebiotics protect against *Salmonella* colonization by competing for the binding sites [18] and increasing the short-chain fatty acids concentrations in the intestine [19].

Intestinal colonization load of *Salmonella* play a role in carcasses contamination at slaughter, hence, reducing *Salmonella* colonization in chickens may potentially reduce salmonellosis incidence in humans [20]. Though extensive studies have been conducted to evaluate the effects of several newly developed and commercially available probiotics on intestinal colonization of *Salmonella* in birds, very little research has been undertaken to identify if the reduced intestinal *Salmonella* colonization translates into decreased carcass contamination. The objective of this study is to identify the effects of four probiotic strains of commercially available synbiotic compound (PoultryStar^®^ ME at 0.5 g/kg feed containing *L*. *reuteri*, *E*. *faecium*, *B*. *animalis*, *P*. *acidilactici and a* prebiotic Fructooligosaccharide) on *Salmonella* proliferation and to identify whether *in vivo* synbiotic supplementation can decrease the *Salmonella* load in the chicken intestine and bacterial load on carcass.

## Materials and methods

All animal protocols were approved by the Institutional Animal Care and Use Committee at the University of Georgia.

### *in vitro* study

#### Cell-free probiotic supernatants on *S*. *Enteritidis in vitro* proliferation

Single isolated colonies of *Lactobacillus reuteri*, *Enterococcus faecium*, *Bifidobacterium animalis*, and *Pediococcus acidilactici* probiotic strains were inoculated into 50 mL of MRS (DeMan-Rogosa-Sharpe; Sigma Aldrich, St Louis, MO, USA) broth and incubated overnight at 37° C. Once the overnight probiotic cultures reached an optical density between 0.9–1.2 at 600 nm wavelength (O.D 600), cultures were centrifuged at 4,500 X g for 10 min and the supernatant was collected. The supernatant was filter-sterilized using 0.22μm filter (EMD Millipore, MA, USA) to collect cell-free supernatant. A primary isolate of *S*. *Enteritidis* [9] was inoculated into 15 mL of Tryptic Soy broth and incubated at 37° C for 12 h.

A volume of 10 μl of *S*. *Enteritidis* overnight culture (O.D 600 = 0.1) was incubated with 0:1, 1:1, 5:1, or 10:1 supernatant: pathogen dilutions in triplicates (n = 3) in 96-well flat-bottom plate. The total incubated volume was adjusted to 110 μl using MRS broth. The 96-well plates were incubated at 37°C for 24 h. After incubation, the absorbance was measured at 600nm and the effect of probiotic culture supernatant inhibition on *Salmonella* proliferation was reported as optical density (OD) values. This assay was conducted in triplicates in three independent experiments (n = 3).

### *in vivo* study

#### Birds and *S*. *Enteritidis* infection

A total of 240 Cobb-500 broiler chicks were randomly allotted to one of three treatment groups, control (basal diet; corn-soybean meal diet), antibiotic (Virginiamycin at 20 mg/Kg feed; Stafac^®^20, Phibro Animal Health, Teaneck, NJ), and synbiotic (Poultrystar^®^ ME^US^ at 0.5g/Kg feed; Biomin America Inc., Overland Park, KS) from day of hatch. Experimental basal feed was a corn-soybean meal diet (Table 1). The synbiotic (PoultryStar^®^ ME, BIOMIN America, Inc.) contained four live strains isolated from adult chickens (*L*. *reuteri*, *E*. *faecium*, *B*. *animalis*, and *P*. *acidilactici*) with the prebiotic, Fructooligosaccharide. Each treatment was replicated in eight floor pens with 10 chicks per pen (n = 8). Chickens had ad libitum access to water and feed during the entire experimental period. Bodyweight and feed consumption were measured at weekly intervals, and body weight gain and feed conversion ratio (FCR) were calculated. At 21 d of age, all birds in experimental groups were inoculated orally with 250 μl of 1 X 10^9^ colony forming units (CFU) of nalidixic acid-resistant *S*. *Enteritidis*, the same strain used for *in vitro* study. The nalidixic acid-resistant variants were used to assess the recovery of *Salmonella* from carcass rinses.

**Table 1:** Primer sequences and PCR conditions for housekeeping genes under study^1^.

Gene name	Primer sequence^1^	Annealing temperature
IL-10	F: 5’- CAGACCAGCACCAGTCATCA-3’ R: 5’- CGAACGTCTCCTTGATCTGC-3’	57.5ºC
IL-1β	F: 5’- CTACACCCGCTCACAGTCCT-3’ R: 5’- TCACTTTCTGGCTGGAGGAG-3’	57.5ºC
TLR-4	F: 5’- ACCTACCCATCGGACACTTG-3’ R: 5’- TGCCTGAGAGGTCAGGTT-3’	60.0ºC
IFNγ	F: 5’- CTGATGGCGTGAAGAAGGTG -3’ R: 5’- CTCCTCTGAGACTGGCTCCTTT -3’	57.4ºC
β-actin	F: 5’- ACCGGACTGTTACCAACACC-3’ R: 5’-GACTGCTGCTGACACCTTCA-3’	57.0ºC

^1^Primer sets F, forward; R, reverse.

#### Effect of synbiotic supplementation on cecal *S*. *Enteritidis* load post-Salmonella infection in broiler birds

On 3, 7, 14, and 21 d post-infection, cecal contents were collected from one bird per pen (eight birds per treatment) and analyzed for *S*. *Enteritidis* load by real-time PCR. Bacterial genomic DNA was isolated as described earlier by [21] with some modifications. Cecal contents (0.2g) were washed two times with 1X PBS. The cell pellet was resuspended in EDTA and treated with 20 mg/ml lysozyme for 30 min at 37°C, followed by treatment with lysis buffer containing 20% SDS and 0.1 mg/ml proteinase K (Sigma Aldrich, St Louis, MO) for 5 min at 80°C. The samples were incubated with 5μL of RNase at 37°C for 30 min. The cell lysate was incubated with 6M sodium chloride on ice for 10 min. The supernatant was collected after centrifugation at 400 X g for 10 min. The DNA in the supernatant was precipitated with isopropanol and washed once in ice-cold ethanol. The DNA pellet was resuspended in TE buffer (10 mM Tris-HCl, 1 mM EDTA, pH 8.0) and stored at -20°C until further use.

The DNA extracted from all the treatment groups was analyzed for *S*. *Enteritidis* load by real-time PCR using *S*. *Enteritidis* specific primers F-GCAGCGGTTACTATTGCAGC and R-CTGTGACAGGGACATTTAGCG [22]. The threshold cycle (Cq) values were determined by CFX software (Bio-Rad, Hercules, CA) when the fluorescence rises exponentially 2-fold above background. The copy numbers of *S*. *Enteritidis* specific was expressed in log units as described previously [23].

#### Effect of synbiotic supplementation on *S*. *Enteritidis* carcass rinsate load post-Salmonella infection in broiler birds

At 21 d post-infection, chickens were removed from feed, but not water, for 8 h prior to slaughter, and one chicken from each pen from each of the eight replications (n = 8) was randomly selected for slaughtering. The chickens were processed at the USDA-ARS, Athens processing facility following standard processing protocols for stunning and bleeding. Three counter-current scald tanks were used for scalding. The scalding tanks temperature was 128°, 130°, and 130° Fahrenheit. Chickens were immersed in each scalding tank for 90 seconds, followed by defeathering for 30 seconds. Chickens from antibiotic treatment group were processed first followed by that in the synbiotic treatment group and control treatment group. Scald tank water was changed, all equipment was cleaned and sanitized before, chickens from each treatment group were processed. After removal of the feathers, head, and hocks, the excess fluid was drained from the carcass, which was then transferred to a sterile bag (Cryovac, Charlotte, NC). A 400 milliliter (ml) volume of sterile buffered peptone water (BPW) (Difco Laboratories) was added into each bag. The sterile carcass bags were shaken in a rocking motion for two minutes. One mL of chicken carcass rinsate from each sample was transferred to a polypropylene culture tube containing 9 mL BPW for *Salmonella* enumeration. A 3-tube most probable number (MPN) technique was used to enumerate the *Salmonella* load in the carcass as described earlier [24]. Briefly, one mL of the chicken carcass rinsate was mixed with 9 mL BPW and incubated at 37°C for 24 h. At 24 h incubation, 0.1 mL of pre-enriched sample was inoculated into 9.9 mL of Rappaport-Vassiliadis broth (Sigma Aldrich, St Louis, MO) for selective enrichment of *Salmonella* and incubated at 42°C for 24 h. 10 μL of the enrichment culture was streaked on Xylose- Lysine- Tergitol 4 (XLT4) agar selective media (Hardy Diagnostics) and incubated at 37 °C for 24 h. The number of CFU *Salmonella*, recovered from each rinse sample was determined by manual counting of colonies. *Salmonella* suspected positive black colonies were resuspended in the PBS for confirmation through real-time PCR using *S*. *Enteritidis* specific primers. Rinse fluid CFU from each rinse sample was converted to Log10 CFU/mL of recovered rinse fluid.

#### Synbiotic supplementation on IL-10, IL-1, TLR-4, and IFNγ mRNA amounts in the cecal tonsils

At 3, 7, 14, and 21 d post-infection, cecal tonsils were collected and analyzed for IL-10 IL-1, Toll-like receptor (TLR-4), and Interferon-γ (IFNγ) mRNA content by real-time PCR. On 3, 7, 14, and 21 d post-infection, one bird per pen from each of the eight replications were randomly chosen for sample collection (n = 8). Total RNA was collected from cecal tonsils and reverse transcribed into cDNA [25]. mRNA content for IL-10, IL-1β, TLR4, and IFNγ were analyzed by real-time PCR (CFX96 Touch Real Time System, BioRad) using SyBr green after normalizing for β-actin mRNA [26]. Primer sequences are provided in Table 1. Fold change from the reference was calculated [27] as ES (Ct Sample)/ER (Ct Reference), where ES and ER are the sample and reference PCR amplification efficiencies as determined by LinRegPCR program [28], and Ct is the threshold cycle. Ct was determined by the CFX software (Biorad, Hercules, CA) when the fluorescence rises exponentially two-fold times above background. The reference group was the control diet group.

#### Statistical analysis

A one-way ANOVA was used to determine the effect probiotic culture supernatant on *Salmonella* growth and the effects of antibiotic and synbiotic supplementation on dependent variables (JMP, SAS Institute Inc., Cary, NC). The averages of plate counts were converted to log CFU/ml and were analyzed using one- way ANOVA. When the main effects were significant (P < 0.05), differences between means were analyzed by Tukey's least-square means comparison.

## Results

### *In vitro* experiment

#### Effect of cell-free probiotic supernatants on *S*. *Enteritidis in vitro* proliferation

**Fig 1:**
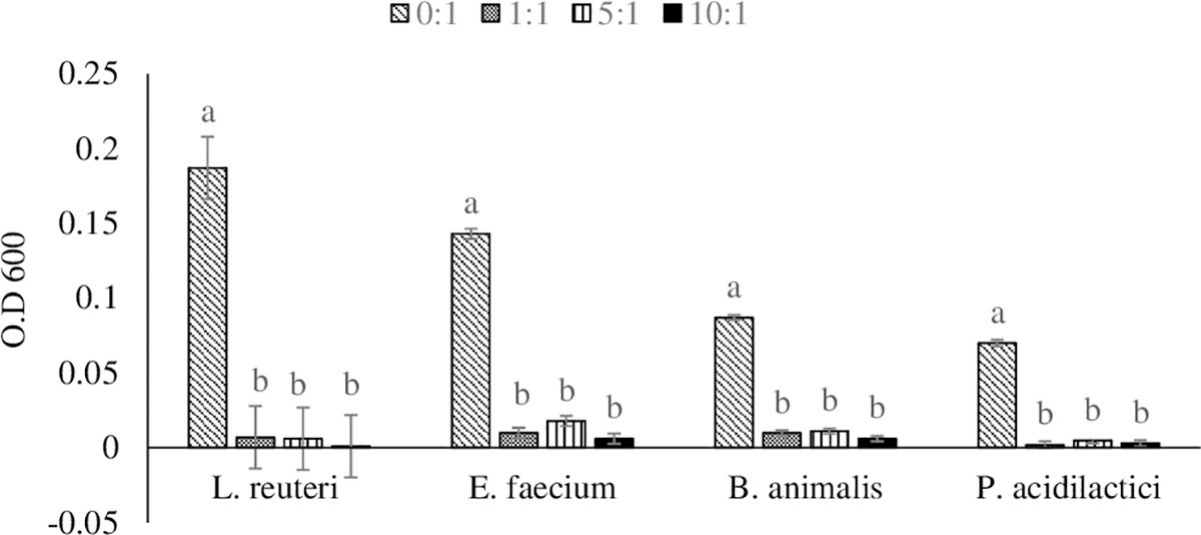
Effect of cell- free probiotic supernatants on *S*. *Enteritidis in vitro* proliferation. Overnight culture of single isolated colonies of *Lactobacillus reuteri*, *Enterococcus faecium*, *Bifidobacterium animalis*, and *Pediococcus acidilactici* probiotic strains were centrifuged at 4,500 X g for 10 min to collect supernatants. The supernatant was filtered using a 0.22μm filter to collect cell-free supernatant. 10 μl of *S*. *Enteritidis* overnight culture was incubated with 0:1, 10:1, 5:1, or 1:1 cell-free supernatant: pathogen dilutions. The absorbance was measured at 600nm at 24 hours. n = 3. Values ± SEM of pooled samples of 3 wells/treatment. Bars (± SEM) with no common superscript (^a, b^) differ significantly (P < 0.05).

All the four probiotics culture supernatants inhibit the growth of *S*. *Enteritidis* at 1:1 supernatant: pathogen dilution compared to the 0:1 dilution group. The proliferation of *S*. *Enteritidis* at 1:1 dilution decreased by 99.5, 95.8, 93.1, and 95.7% for *L*. *reuteri*, *E*. *faecium*, *B*. *animalis*, and *P*. *acidilactici*, respectively, when compared to the 0:1 dilution group. The inhibition at 5:1 dilution was 96.8, 87.4, 87.4, and 92.9% for *L*. *reuteri*, *E*. *faecium*, *B*. *animalis*, and *P*. *acidilactici*, respectively, when compared to the 0:1 dilution group. While the inhibition at 10:1 dilution was 96.3, 93.0, 88.8, and 97.1% for *L*. *reuteri*, *E*. *faecium*, *B*. *animalis*, and *P*. *acidilactici*, respectively, when compared to the 0:1 dilution group (Fig 1).

### *In vivo* experiment

#### Effect of synbiotic supplementation on production parameters in post-*Salmonella* challenge

Synbiotic supplementation had no significant effects on body weight and feed consumption at 21 d and 42 d of age (P > 0.05) compared to control groups (Table 2). At 21 d and 42 d of age antibiotic supplementation had significantly increased BW and feed consumption (P < 0.01) compared to the control groups. Antibiotic and synbiotic supplementation increased BW by 230 and 170 g and feed consumption by 270 and 200 g compared to the control *S*. *Enteritidis* challenge group at 42 d of age, respectively. Synbiotic supplementation had no significant effects on feed conversion ratio at 21 (P = 0.12) and 42 d of age (P = 0.16) compared to control groups in response to *S*. *Enteritidis* challenge.

**Table 2:** Effect of synbiotic supplementation on production parameters post-Salmonella infection in broiler birds.

	0-21d	0-42d
**Body weight (kg)**		
Control	0.79^b^	2.65^b^
Virginiamycin	0.89^a^	2.89^a^
Synbiotic	0.82^b^	2.82^ab^
SEM	0.01	0.05
P value	P < 0.01	P = 0.01
**Feed consumption (kg)**		
Control	1.08^b^	4.47^b^
Virginiamycin	1.17^a^	4.74^a^
Synbiotic	1.10^b^	4.67^ab^
SEM	0.02	0.06
P value	0.01	0.01
**Feed conversion ratio**		
Control	1.37	1.69
Virginiamycin	1.32	1.64
Synbiotic	1.33	1.66
SEM	0.02	0.02
P value	0.12	0.16

Birds were fed either basal diet (Control) or supplemented with 20 mg/Kg feed Virginiamycin (antibiotic) or 0.05% synbiotic product (Poultrystar^®^ ME; Biomin America Inc) day-of-hatch through 42d of age. At 21 d of age, birds were challenged with 1 X 10^9^ CFU of *Salmonella enterica* ser. Enteritidis. Means with no common superscript (^a, b, ab^) within a column differ significantly (P < 0.05). n = 8.

#### Effect of synbiotic supplementation on *S*. *Enteritidis* load in the cecal content post-*Salmonella* challenge

**Fig 2:**
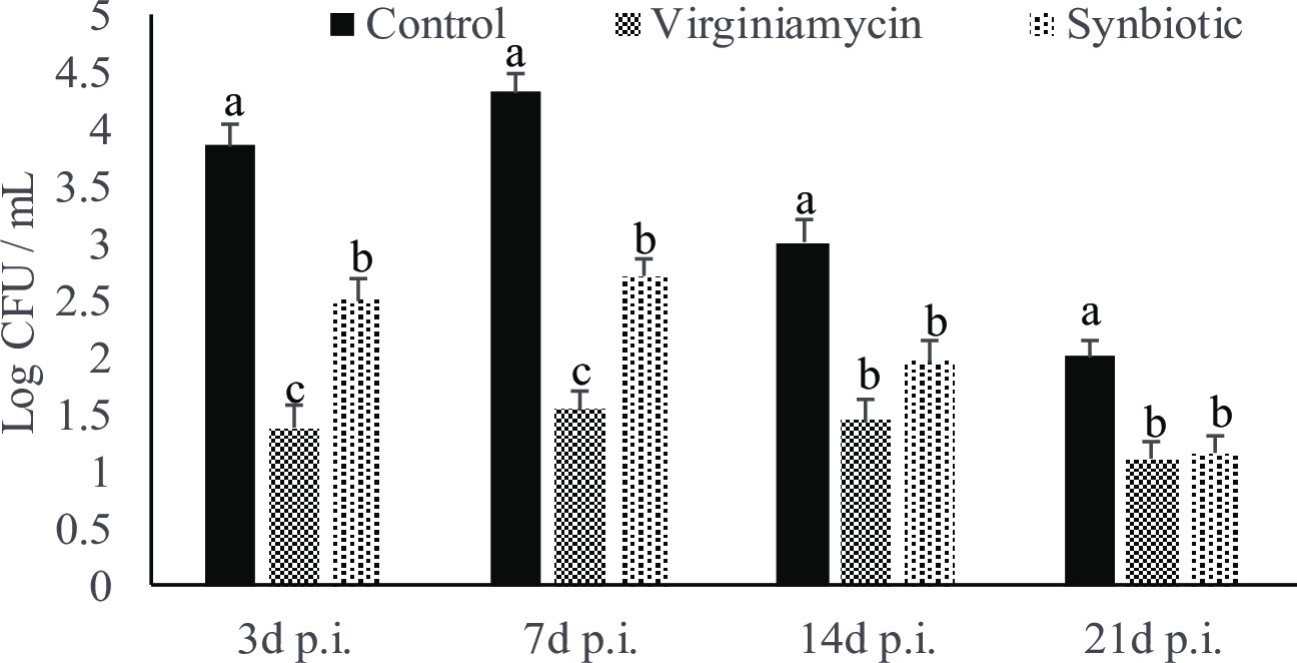
Effect of synbiotic supplementation on *S*. *Enteritidis* load in the cecal content post-*Salmonella* infection in broilers. Birds were fed either basal diet (Control) or supplemented with 20 mg/Kg feed Virginiamycin (antibiotic) or 0.05% synbiotic product from day-of-hatch through 42d of age. At 21 d of age, birds were challenged with 1 X 10^9^ CFU of *S*. *Enteritidis*. At 3, 7, 14, and 21 d post-infection, cecal content were analyzed for *S*. *Enteritidis* load by real-time PCR collected and expressed as log values. Bars (± SEM) with no common superscript (^a, b, c^) differ significantly (P < 0.05). n = 8.

Antibiotic and synbiotic supplementation had significant effects on *S*. *Enteritidis* load in the cecal content at 3 (P < 0.01), 7 (P < 0.01), 14 (P < 0.01), and 21 (P = 0.01) d post-infection (Fig 2). Antibiotic and synbiotic supplementation decreased *S*. *Enteritidis* load in the cecal content at by 0.90 and 0.85 log units, respectively compared to the control group at 21 d post-*Salmonella* infection.

#### Effect of synbiotic supplementation on carcass *S*. *Enteritidis* load post- *Salmonella* challenge

**Fig 3:**
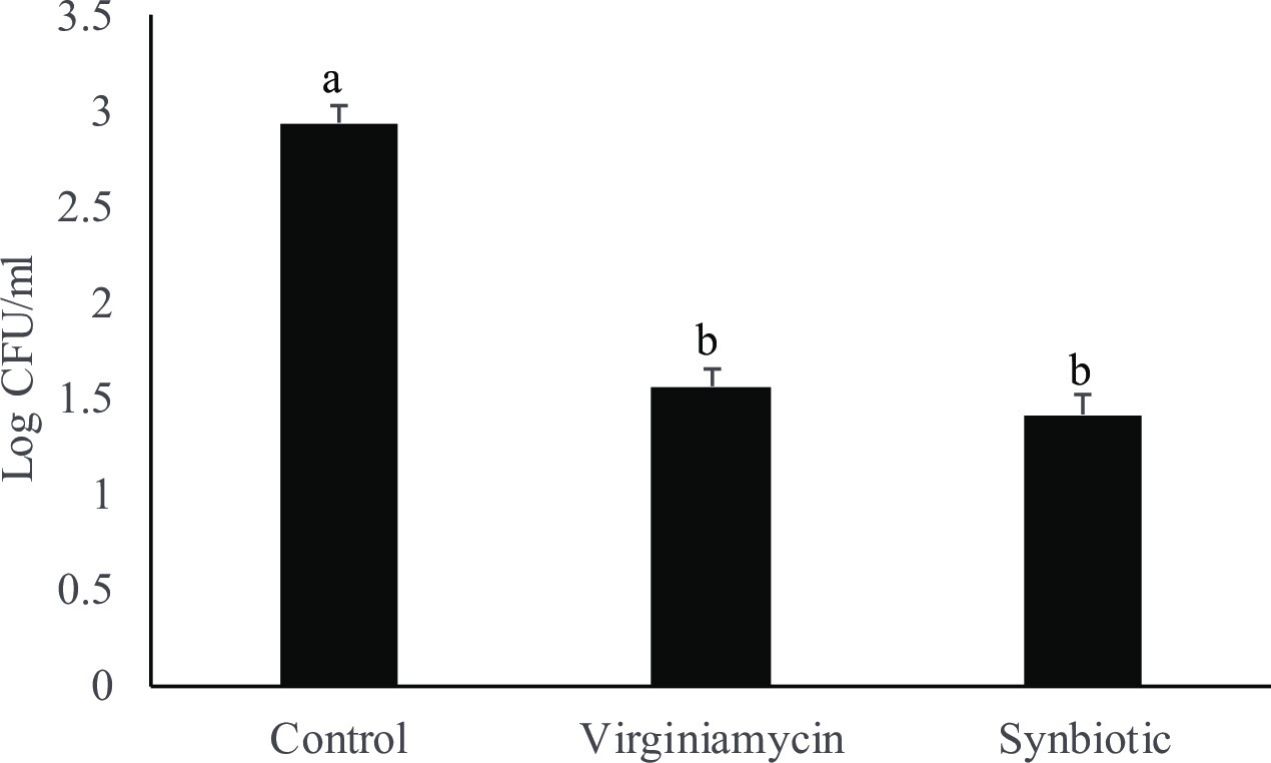
Effect of synbiotic supplementation on chilled carcass *S*. *Enteritidis* rinsate load post-Salmonella infection in broiler birds. Birds were fed either basal diet (Control) or supplemented with 20 mg/Kg feed Virginiamycin (antibiotic) or 0.05% synbiotic product from day-of-hatch through 42d of age. At 21 d of age, birds were challenged with 1 X 10^9^ CFU of *S*. *Enteritidis*. At 21 d post-infection, the carcass *S*. *Enteritidis* most probable number (MPN) was analyzed by plating on XLT agar and expressed as log MPN/ml. Bars (± SEM) with no common superscript (^a, b^) differ significantly (P < 0.05). n = 8.

Antibiotic and synbiotic supplementation had significant effects on chilled carcass *S*. *Enteritidis* load at 21 d (P = 0.02) post-infection (Fig 3). Antibiotic and synbiotic supplementation decreased carcass *S*. *Enteritidis* rinsate load at by 1.4 and 1.5 log units, respectively compared to the control group at 21 d post-*Salmonella* infection.

#### Effect of synbiotic supplementation on cecal tonsil gene expression post-*Salmonella* challenge

**Fig 4:**
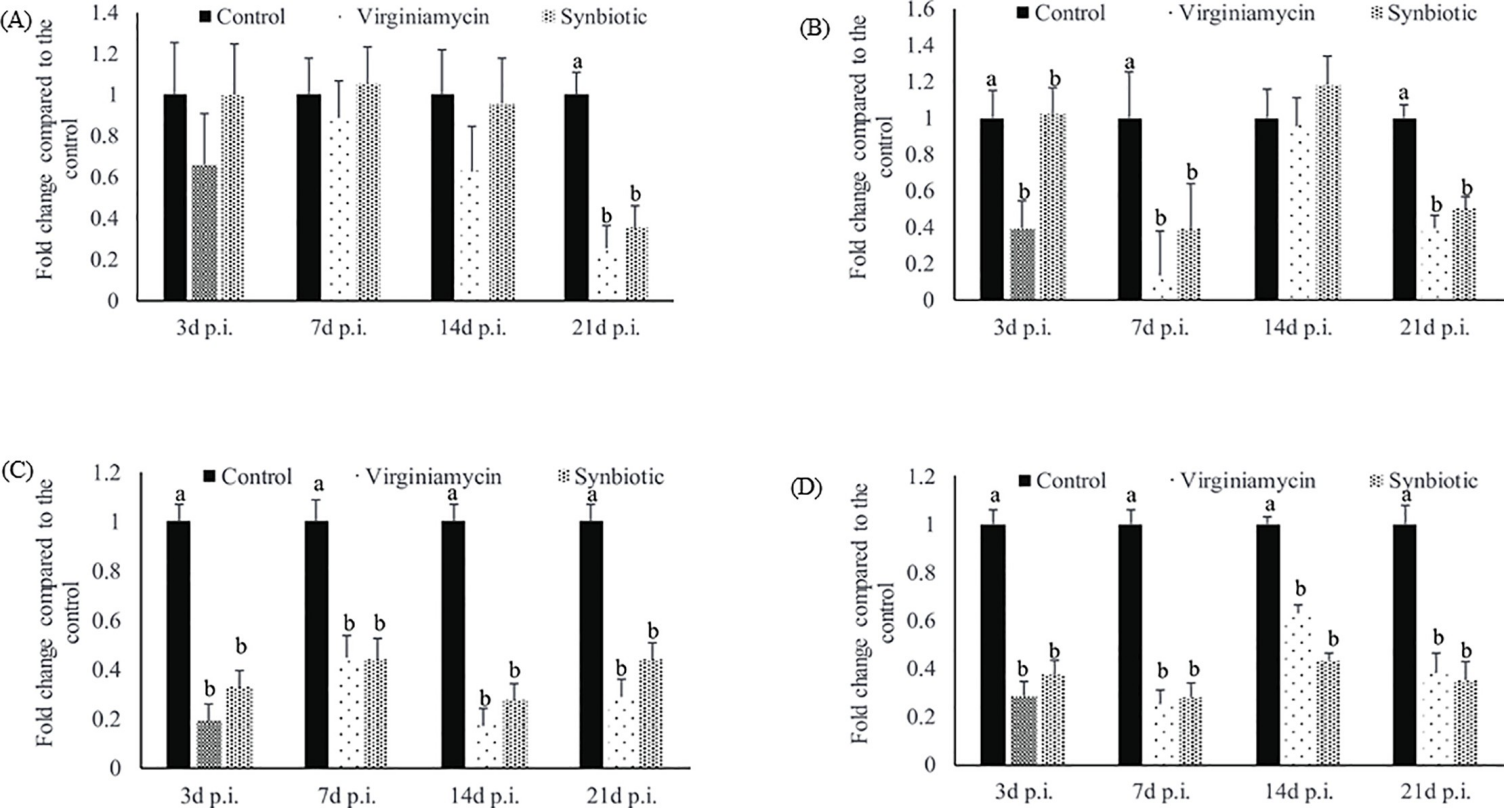
Effect of synbiotic supplementation on cecal tonsil mRNA content post-Salmonella infection in broilers. Birds were fed either basal diet (Control) or supplemented with 20 mg/Kg feed Virginiamycin (antibiotic) or 0.05% synbiotic product from day-of-hatch through 42d of age. At 21 d of age, birds were challenged with 1 X 10^9^ CFU of *S*. *Enteritidis*. At 3, 7, 14 and 21 d post-infection (p.i.), relative IL-10 (Fig 4A), IL-1 (Fig 4B), TLR-4 (Fig 4C), and IFN γ (Fig 4D) mRNA content in cecal tonsils were analyzed from 1 bird/pen; 8 pens/diet after correcting for β-actin mRNA and normalizing to the mRNA content of the control group. Bars (± SEM) with no common superscript (^a, b^) differ significantly. n = 8.

Antibiotic and synbiotic supplementation reduced cecal tonsil IL-10 mRNA content at 21 (P < 0.01) d post-infection (Fig 4A).

Antibiotic and synbiotic supplementation had significant effects on cecal tonsil IL-1 mRNA content at 3 (P = 0.01), 7 (P = 0.01), and 21 (P < 0.01) d post-infection (Fig 4B). At 21 d post-infection, birds in the antibiotic and synbiotic supplementation group had 60, and 50% decreased IL-1 mRNA compared to the control group, respectively.

Antibiotic and synbiotic supplementation had significant effects on cecal tonsil TLR-4 mRNA content at 3 (P < 0.01), 7 (P < 0.01), 14 (P <0.01) and 21 (P < 0.01) d post-infection (Fig 4C). At 21 d post-infection, birds in the antibiotic and synbiotic supplementation group had 70, and 56% decreased TLR-4 mRNA compared to the control group, respectively.

Antibiotic and synbiotic supplementation had significant effects on cecal tonsil IFNγ mRNA content at 3 (P < 0.01), 7 (P < 0.01), 14 (P < 0.01) and 21 (P < 0.01) d post-infection (Fig 4D). At 21 d post-infection, birds in the antibiotic and synbiotic supplementation group had by 0.61, and 0.65% decreased IFNγ mRNA compared to the control group, respectively.

## Discussion

This study identified that all four probiotics strains supernatants had decreased *in vitro* proliferation of *S*. *Enteritidis* separately, and synbiotic supplementation from the day of hatch decreased the *Salmonella* load in the chicken cecal contents and decreased carcass contamination in broiler birds.

Supernatants from probiotic strains *L*. *reuteri*, *P*. *acidilactici*, *B*. *animalis* and *E*.*faecium* decreased the proliferation of *S*. *Enteritidis in vitro*. Our results are consistent with previous studies conducted with Lactic acid bacteria (LAB) and Enterococcus bacteria. LAB produce antimicrobial substances such as organic acids, bacteriocins [29], and peptidoglycan hydrolases [30] which can be expected to decrease *Salmonella* proliferation. In addition, LAB have been shown to have a competitive advantage over pathogenic microorganism in the gut because LAB can tolerate low intestinal pH and bile [31]. *L*. *reuteri* exhibits inhibitory effects against both *S*. *Enteritidis* and *S*. *Typhimurium* [32]. Similarly, *E*. *faecium* and *P*. *acidilactici* produce enterocins and pediocins respectively, which have been shown to inhibit the growth of gram-positive and gram-negative pathogenic bacteria [33]. *B*. *animalis* produces lactic acid and other bactericidal substances to inhibit the growth of *Salmonella* [34]. Supernatants from all four probiotic strains efficiently inhibited the proliferation of *S*. *Enteritidis*, suggesting that *in vivo* supplementation of these probiotic strains to the chickens might be beneficial during a *Salmonella* infection.

In this study, we demonstrated that *in vivo* synbiotic supplementation from the day of hatch decreased the *S*. *Enteritidis* load in the cecal tonsils and decreased carcass contamination in broiler birds. Our laboratory has previously shown that *L*. *reuteri*, *P*. *acidilactici*, *B*. *animalis*, and *E*.*faecium* can successfully colonize the chicken intestine [23]. The consistent effect of the probiotics in decreasing the proliferation of *S*. *Enteritidis* both *in vitro* and *in vivo* suggest that probiotics will be a major tool in combating *Salmonella* load in birds.

In this study, chickens fed synbiotics and antibiotics had decreased *Salmonella* load in both cecal content and carcass rinsate. In pigs, synbiotics decrease *Salmonella* loads in the intestine and decrease *Salmonella* contamination of carcasses which suggest that decreasing intestinal *Salmonella* load in the intestine would be the ideal approach to decrease carcass load [35]. In chickens, *Salmonella* can colonize the gut efficiently and thereby can be shed in the feces for an extended period without showing symptoms. *Salmonella*-contaminated feces play a major role in carcass contamination and horizontal transmission in chicken [36].; Probiotics which act against gut microbes through competitive exclusion treatments has been shown to reduce *Salmonella* flock prevalence by up to 70–85% [37]. In our study, synbiotic supplementation decreased the *Salmonella* load in both cecal content and carcass rinsate suggesting that synbiotics not only efficiently colonized the intestine but also secreted antibacterial substances in the gut lumen to decrease the *S*. *Enteritidis* load in the carcass.

TLR-4 is a pattern recognition receptor and recognizes the lipopolysaccharide of gram-negative bacteria and is an indicator of immune stimulation following *Salmonella* infection [38]. Chickens in the antibiotic and synbiotic supplemented groups had decreased cecal tonsil TLR-4 mRNA content compared to the control groups post-*Salmonella* infection throughout the study.

Synbiotics may protect against *Salmonella* infection through different mechanisms, including modulation of cytokine responses. Probiotic bacteria such as *E*. *faecium* and *L reuteri* exert immunomodulatory activities by altering the host cytokine expression profiles [39, 40]. *Salmonella* can also stimulate the host immune cells to modify host cytokines and chemokines [40]. IFNγ is an inflammatory cytokine that acts to improve the host defense against intracellular pathogen like *Salmonella*. Chickens in the control group infected with *Salmonella* had higher IFNγ compared to the *Salmonella*-infected birds fed antibiotic and synbiotic all-time points of this study. Considering that supplementation of synbiotic and antibiotic in birds challenged with *Salmonella* decreased *Salmonella* load in the cecal content, the decreased *Salmonella* load likely contributed to the decreased IFNγ mRNA in the cecal tonsil. *Salmonella* infection increase IL-10 mRNA content in the cecal tonsils [41]. IL-10 is a regulatory cytokine produced by T regulatory cells, and upregulation of IL-10 by *Salmonella* is a pathogen defense mechanism to create a persistent infection of chickens [9]. Synbiotic and antibiotic supplementation reduced *Salmonella* load in the cecal content of *Salmonella* infected birds and the reduced *Salmonella* load likely contributed to the decreased IL-10 mRNA in the cecal tonsil.

In conclusion, our study confirmed that the *in vivo s*ynbiotic supplementation improves the body weight and feed intake and reduce the colonization of *Salmonella* in the cecal content of broiler chickens. Therefore, administration of synbiotics can reduce or replace the use of antibiotics in poultry production and reduce the incidence of *Salmonella* load in the carcass.
